# Risk of cancer-specific, cardiovascular, and all-cause mortality among Asian and Pacific Islander breast cancer survivors in the United States, 1991–2011

**DOI:** 10.1186/s40064-016-1726-3

**Published:** 2016-01-26

**Authors:** Pooja A. Solanki, Naomi Y. Ko, Dima M. Qato, Gregory S. Calip

**Affiliations:** Department of Epidemiology and Biostatistics, University of Illinois at Chicago, 1603 W. Taylor St., Chicago, IL 60612 USA; Section of Hematology Oncology, Boston University, Boston Medical Center, 725 Albany St., Boston, MA 02118 USA; Center for Pharmacoepidemiology and Pharmacoeconomic Research, University of Illinois at Chicago, 833 S. Wood St., Chicago, IL 60612 USA; Population Health, Behavior and Outcomes Program, University of Illinois Cancer Center, 1801 W. Taylor St., Chicago, IL 60612 USA

**Keywords:** Breast cancer, Asian American, Pacific Islander, Cancer survivorship, Mortality

## Abstract

Asian and Pacific Islander (API) women in the United States (U.S.) are a heterogeneous group reported to have better prognosis after breast cancer (BC) compared to their Non-Hispanic White (NHW) counterparts. Few studies have examined differences in BC survival between individual API ethnic groups. We conducted a retrospective cohort study of 462,005 NHW and 44,531 API women diagnosed with incident, stage I–III BC between 1991 and 2011 in the Surveillance, Epidemiology and End Results (SEER) 18 registries. SEER-reported API ethnicity was grouped as Chinese, Japanese, Filipino, Hawaiian, Korean, Vietnamese, Asian Indian and Pakistani, and Pacific Islander. Multivariable Cox proportional hazards models were used to estimate hazard ratios (HR) and 95 % confidence intervals (CI) for risk of BC-specific, cardiovascular and all-cause mortality comparing API to NHW women. We also estimated mortality risk comparing U.S.-born to non-U.S.-born women. Compared to NHW women, API women overall had lower BC-specific, cardiovascular and all-cause mortality. BC-specific mortality risk was lowest among Japanese women (HR 0.69, 95 % CI 0.63–0.77). Other women had similar (Filipino, HR 0.93, 0.86–1.00; Hawaiian, HR 1.01, 0.89–1.17) or greater (Pacific Islander, HR 1.44, 1.17–1.78) risk of BC-specific death. Compared to non-U.S. born API women, findings were suggestive of increased cardiovascular (HR 1.12, 1.03–1.20) and all-cause mortality (HR 1.29, 1.08–1.54) among U.S.-born API women. Mortality risk varies greatly between BC survivors from different API backgrounds. Further research is warranted to understand these disparities in BC survivorship and the social and cultural factors that possibly contribute to greater mortality among later-generation API women born in the United States.

## Background

Current understanding of breast cancer epidemiology among Asian women is limited due to the aggregation of data on Asian race and ethnicity. Asian and Pacific Islander (API) communities compose the fastest growing racial/ethnic group in the United States (U.S.) (U.S. Census Bureau 2012). This group originates from the earth’s largest continent and encompasses a heterogeneous population with respect to multiple races, immigration status, religion, specific cultural factors, health beliefs and health practices, including those related to cancer and cancer screening (Thompson et al. 2014; Sabatino et al. 2015; Kagawa-Singer and Pourat 2000; Gomez et al. 2004, 2007).

Breast cancer (BC) is the most frequently diagnosed cancer in women. Despite overall declines in BC over the past 5–10 years in other U.S. racial and ethnic groups, incidence rates among some but not all API ethnic groups show increasing trends (American Cancer Society 2013). API women overall are reported to have the highest 5-year survival rates after breast cancer (91.4 %), but these data are unreliable given this large heterogeneous group. Even less is known about long-term outcomes among API breast cancer survivors in the U.S.

Across the Asian continent, cancer outcomes vary greatly by country and ethnicity. In a global surveillance assessment in 2010, the 5-year breast cancer survival rates in some Asian countries, including Japan, China, Taiwan, Indonesia, Korea, and Thailand, were greater than 80 %. However, in India, survival rates are reported to be less than 60 % (Allemani et al. 2015). The aggregation of these populations living in the U.S. into one group dilutes differences between distinct API ethnic groups that could explain differential cancer-specific mortality, cardiovascular mortality and all-cause mortality in API breast cancer survivors (Wu et al. 2013; Lin et al. 2002; Trinh et al. 2015). In prior studies of API women in the U.S., greatest survival is reported among Japanese women (Allemani et al. 2015), whereas Hawaiian and Filipino women experience poor breast cancer survival after diagnosis compared to NHW women (Meng et al. 1997; Hsu et al. 1997).

Few studies exist on differences in long-term survival after breast cancer by specific API ethnic groups in the U.S. Studies on cancer outcomes among Asian Americans tend to be limited by small sample sizes or represent only geographic areas in the U.S. with large API populations such as California. The collection of data on cancer incidence and follow up with information on specific API ethnic groups by U.S. population-based cancer registries in the Surveillance, Epidemiology and End Results (SEER) Program will increase our understanding of the heterogeneity of breast cancer in API women.

Our objective was to disaggregate the large and heterogeneous “Asian” group to determine differences in the risk of breast cancer-specific, cardiovascular and all-cause mortality among breast cancer survivors by API ethnicity. We further examined possible differences in mortality risk among API women born in the U.S. compared to non-U.S. born women.

## Methods

### Study population and data sources

We conducted a retrospective cohort study of women diagnosed with a first primary invasive, stage I–III breast cancer between January 1, 1991 and December 31, 2011 identified through twelve population-based cancer registries in the U.S. that participate in the National Cancer Institute’s SEER Program [Surveillance Epidemiology and End Results (SEER) Program (2013)]—those serving the geographic areas of San Francisco-Oakland, Connecticut, Detroit, Hawaii, Iowa, New Mexico, Seattle-Puget Sound, Utah, Atlanta, San Jose-Monterey, Los Angeles, Rural Georgia, California (excluding SF/SJM/LA), Kentucky, Louisiana, New Jersey and Greater Georgia. SEER ascertains and follows demographic and tumor-specific information including age at diagnosis, stage and vital status with detail on cause-specific death. Further details and methods used by the SEER Program are provided elsewhere (Lash et al. 2007).

A total of 462,005 NHW and 44,531 API women ages 20 years and older with invasive, stages I–III BC documented in SEER as their first primary cancer diagnosis (no history of cancer before index diagnosis) that received cancer-directed surgery were included in this study. Women were excluded if their cancer was diagnosed at autopsy and if their race was classified as other or unknown.

The primary exposure of interest was race and ethnicity. We used SEER-coded categories of race and ethnicity to classify study participants. We used these categorizations in our analyses to classify women into mutually exclusive categories of Non-Hispanic White and Asian/Pacific Islander. API ethnicity was disaggregated into groups reported as Chinese, Japanese, Filipino, Hawaiian, Korean, Vietnamese, Asian Indian and Pakistani, Pacific Islander and Other Asian. Pacific Islander women included those with ethnicity reported as Micronesian, Chamorran, Guamanian NOS, Polynesian NOS, Tahitian, Samoan, Tongan, Melanesian NOS, Fiji Islander, New Guinean, and Other Pacific Islander. For purposes of small reported number of cases, women grouped as ‘Other Asian’ included Thai, Laotian, Hmong, Kampuchean and unspecified/other.

We ascertained data for this cohort of BC survivors from the SEER registries, including: age at diagnosis, year of diagnosis, American Joint Committee on Cancer (AJCC) stage (Singletary et al. 2003), estrogen receptor (ER) status, progesterone receptor (PR) status, tumor size, and lymph node status. Information on type of surgery and receipt of radiation treatment was obtained, but data on adjuvant chemotherapy and adjuvant hormonal therapy were not available. Data regarding other socioeconomic factors, such as income and health insurance status, were also not available. Women included were required to have cancer-directed surgery documented in SEER, classified as mastectomy (radical or total) or breast-conserving surgery (less than total mastectomy: segmental mastectomy, lumpectomy, quadrectomy, tylectomy, wedge resection, nipple resection, excisional biopsy, or partial mastectomy NOS).

For a secondary analysis, we obtained SEER-documented place of birth for women as U.S. born, non-U.S. born and birthplace not specified or missing. Because the exposure of interest was U.S. born status within API race/ethnicity, we compared U.S.-born women to non-U.S. born, although this analysis was limited by unknown place of birth for 16,150 (36.3 %) of API women.

Survival time was calculated in months from diagnosis to the date of death or to the date last known to be alive. The closure date for follow-up observation was December 31, 2012. The outcomes in these analyses, death due to breast cancer and death due to cardiovascular causes, were examined separately to account for competing causes of death which differed by ethnic group (Howlader et al. 2010). We also examined all-cause mortality without considering these competing risks. Breast cancer cases were considered to have experienced breast cancer-specific mortality if their cause of death was classified by International Classification of Diseases, Ninth Revision (ICD-9) codes as death due primary malignant breast cancer. The outcome of cardiovascular-specific mortality was similarly defined using ICD-9 codes for death due to diseases of the heart, hypertension without heart disease, cerebrovascular disease, atherosclerosis, aortic aneurysm and dissection and other diseases of arteries, arterioles and capillaries. Cases who died from other causes or were lost to follow-up were considered censored at the time of loss or death in cause-specific mortality analyses (Varadhan et al. 2010).

Survival analyses were performed using Cox proportional hazards models to calculate adjusted hazard ratio (HRs) and 95 % confidence intervals (CIs) for risk of breast cancer-specific, cardiovascular and all-cause mortality comparing women of specific API ethnicities to NHW women. Mortality risks were estimated in models adjusting for year of diagnosis, SEER registry, age (20–49, 50–64, 65–74, 75–84, 85+ years) and AJCC stage (I, II, III) and in fully multivariable-adjusted models that included adjustment for confounding variables selected a priori, including, birthplace (U.S., non-U.S., unknown), marital status (single or never married, married or domestic partner, unknown), hormone receptor status (ER+/PR+, ER+/PR−, ER−/PR+, ER−/PR−, unknown), tumor size (<2, ≥2 cm, unknown), lymph node status (negative, positive, unknown), surgical procedure (radical or total mastectomy, breast-conserving surgery, unspecified surgery) and radiation (yes/no). Separate analyses within each API ethnicity examined the effect of U.S. birthplace by comparing the survival of women born in the U.S. to women not born in the U.S.

We evaluated proportional hazards assumptions by plotting the logarithm of the negative logarithm of the survival function over time. No evidence of violation of these assumptions was found. All analyses were performed using SAS statistical software version 9.3 (Cary, NC).

### Ethics, consent and permissions

Approval was obtained from the Institutional Review Board at the University of Illinois at Chicago for this data analysis. No patient identifiers were included. Because information from SEER*Stat is de-identified and based on cancer registry data, it is not possible to seek informed consent from each participant, and we therefore received a waiver of consent.

## Results

Descriptive characteristics of the 462,005 NHW and 44,531 API women diagnosed between 1991 and 2011 that were included in our analysis are reported in Table 1. Median age at diagnosis was higher among NHW women compared to API women overall. API women were more often non-U.S. born and had slightly greater proportions of women diagnosed with BC that was AJCC stage II or III, ER(−)/PR(−), and have positive lymph node status. More API women received radical or total mastectomy versus breast-conserving surgery. Among overall crude death rates by the end of follow-up, 23.8 % of NHW women death due to any cause of which 42.0 % were due to breast cancer and 23.2 % were due to cardiovascular disease. Overall, 15.4 % of API women experienced death due to any cause of which 53.3 % were breast cancer-specific and 17.1 % were due to cardiovascular disease. Descriptive characteristics of API women by ethnic group are reported in Table 2. Characteristics varied considerably by API ethnicity with median age at BC diagnosis being lowest among Korean, Vietnamese, Asian Indian and Pakistani women. Among API groups, Asian Indian and Pakistani women and Pacific Islander women had higher proportions of stage III BC at diagnosis (17.9 and 20.7 %, respectively) compared to the proportion of NHW women diagnosed with stage III BC (12.8 %).

**Table 1:** Descriptive characteristics of Non-Hispanic White and Asian/Pacific Islander women with stages I–III breast cancer in the SEER 18 registries, 1991–2011

	Non-Hispanic White women (n = 462,005)	Asian/Pacific Islander^a^ women (n = 44,531)
Age at index breast cancer		
Mean (SD)	61.2 (13.7)	56.3 (13.1)
Median (IQR)	61 (51–72)	55 (46–66)
20–49 years	102,558 (22.2)	15,241 (34.2)
50–64 years	168,817 (36.5)	17,111 (38.4)
65–74 years	100,839 (21.8)	7683 (17.3)
75–85 years	70,659 (15.3)	3789 (8.5)
85+ years	19,132 (4.1)	707 (1.6)
Years of diagnosis		
1991–1995	57,530 (12.5)	4646 (10.4)
1996–2000	84,289 (18.2)	8009 (18.0)
2001–2005	145,993 (31.6)	12,856 (28.9)
2006–2011	174,193 (37.7)	19,020 (42.7)
Birthplace		
U.S.	194,905 (42.2)	8424 (18.9)
Non-U.S.	20,923 (4.5)	19,957 (44.8)
Unknown	246,177	16,150
Marital status		
Single or never married	47,135 (10.2)	5372 (12.1)
Married or domestic partner	400,184 (86.6)	38,178 (85.7)
Unknown	14,686	981
AJCC stage		
I	242,188 (52.4)	21,664 (48.6)
II	160,716 (34.8)	16,855 (37.9)
III	59,101 (12.8)	6012 (13.5)
Hormone receptor status		
ER(+)/PR(+)	282,720 (61.2)	26,974 (60.6)
ER(+)/PR(−)	50,909 (11.0)	4623 (10.4)
ER(−)/PR(+)	6931 (1.5)	823 (1.8)
ER(−)/PR(−)	71,453 (15.5)	7668 (17.2)
Missing	49,992	4443
Tumor size		
<2 cm	365,185 (79.0)	35,248 (79.2)
≥2 cm	94,405 (20.4)	9097 (20.4)
Missing	2415	186
Lymph node status		
Negative	315,405 (68.3)	29,897 (67.1)
Positive	146,516 (31.7)	14,625 (32.8)
Missing	84	9
Surgery		
Radical or total mastectomy	199,975 (43.3)	22,558 (50.7)
Breast-conserving surgery	223,197 (48.3)	19,109 (42.9)
Unspecified surgery	38,833	2864
Radiation		
No	213,565 (46.2)	21,825 (49.0)
Yes	235,859 (51.1)	21,563 (48.4)
Unknown	12,581	1143
Person-years of follow-up		
Mean (SD)	6.8 (4.9)	6.7 (5.0)
Median (IQR)	4 (2–6)	3 (2–5)
<2 years	80,086 (17.3)	8754 (19.7)
2–4 years	79,702 (17.3)	7831 (17.6)
4–6 years	70,179 (15.2)	6605 (14.8)
6–8 years	60,933 (13.2)	5566 (12.5)
8–10 years	54,674 (11.8)	4700 (10.6)
10+ years	116,431 (25.2)	11,075 (24.9)
Outcome status		
Alive	352,200 (76.2)	37,658 (84.6)
Died (all causes)	109,805 (23.8)	6873 (15.4)
Breast cancer-specific	46,116 (42.0)	3661 (53.3)
Cardiovascular	25,527 (23.2)	1173 (17.1)

^a^Asian/Pacific Islander includes women classified in SEER as Chinese, Japanese, Filipino, Hawaiian, Korean, Vietnamese, Laotian, Hmong, Kampuchean, Thai, Asian Indian, Pakistani, Micronesian, Chamorran, Guamanian NOS, Polynesian NOS, Tahitian, Samoan, Tongan, Melanesian NOS, Fiji Islander, New Guinean, Other Asian and Other Pacific Islander

**Table 2:** Descriptive characteristics of women with stages I–III breast cancer by Asian/Pacific Islander ethnicity in the SEER 18 registries, 1991–2011

	Non-Hispanic White women (n = 462,005)	Chinese (n = 8416)	Japanese (n = 7858)	Filipino (n = 11,193)	Hawaiian (n = 2569)
Age at index breast cancer					
Mean (SD)	61.2 (13.7)	55.9 (13.5)	61.7 (13.6)	56.0 (12.1)	57.0 (12.5)
Median (IQR)	61 (51–72)	54 (46–66)	62 (51–72)	55 (47–64)	57 (48–66)
20–49 years	102,558 (22.2)	3164 (37.6)	1719 (21.9)	3628 (32.4)	761 (29.6)
50–64 years	168,817 (36.5)	2972 (35.3)	2635 (33.5)	4793 (42.8)	1088 (42.4)
65–74 years	100,839 (21.8)	1361 (16.2)	1937 (24.7)	1903 (17.0)	490 (19.1)
75–85 years	70,659 (15.3)	746 (8.9)	1299 (16.5)	753 (6.7)	201 (7.8)
85+ years	19,132 (4.1)	173 (2.1)	268 (3.4)	116 (1.0)	29 (1.1)
Birthplace					
U.S.	194,905 (42.2)	930 (11.1)	3838 (48.8)	670 (6.0)	2078 (80.9)
Non-U.S.	20,923 (4.5)	4398 (52.3)	1423 (18.1)	7339 (65.6)	22 (0.9)
Unknown	246,177	3088	2597	3184	469
Marital status					
Single or never married	47,135 (10.2)	994 (11.8)	972 (12.4)	1429 (12.8)	386 (15.0)
Married or domestic partner	400,184 (86.6)	7252 (86.2)	6772 (86.2)	9523 (85.1)	2131 (83.0)
Unknown	14,686	170	114	241	52
AJCC stage					
I	242,188 (52.4)	4250 (50.5)	4624 (58.8)	5022 (44.9)	1221 (47.5)
II	160,716 (34.8)	3128 (37.2)	2484 (31.6)	4442 (39.7)	959 (37.3)
III	59,101 (12.8)	1038 (12.3)	750 (9.5)	1729 (15.4)	389 (15.1)
Hormone receptor status					
ER(+)/PR(+)	282,720 (61.2)	5059 (60.1)	5083 (64.7)	6572 (58.7)	1775 (69.1)
ER(+)/PR(−)	50,909 (11.0)	869 (10.3)	842 (10.7)	1226 (11.0)	224 (8.7)
ER(−)/PR(+)	6931 (1.5)	152 (1.8)	178 (2.3)	190 (1.7)	65 (2.5)
ER(−)/PR(−)	71,453 (15.5)	1445 (17.2)	1062 (13.5)	1977 (17.7)	371 (14.4)
Missing	49,992	891	693	1228	134
Tumor size					
<2 cm	365,185 (79.0)	6685 (79.4)	6259 (79.7)	8534 (76.2)	1911 (74.4)
≥2 cm	94,405 (20.4)	1695 (20.1)	1571 (20.0)	2613 (23.3)	651 (25.3)
Missing	2415	36	28	46	7
Lymph node status					
Negative	315,405 (68.3)	5698 (67.7)	5890 (75.0)	7299 (65.2)	1701 (66.2)
Positive	146,516 (31.7)	2717 (32.3)	1967 (25.0)	3893 (34.8)	867 (33.7)
Missing	84	1	1	1	1
Surgery					
Radical or total mastectomy	199,975 (43.3)	4301 (51.1)	3425 (43.6)	6299 (56.3)	1115 (43.4)
Breast-conserving surgery	223,197 (48.3)	3510 (41.7)	3536 (45.0)	4326 (38.6)	1180 (45.9)
Unspecified surgery	38,833	605	897	568	274
Radiation					
No	213,565 (46.2)	4185 (49.7)	3497 (44.5)	5797 (51.8)	1049 (40.8)
Yes	235,859 (51.1)	4015 (47.7)	4218 (53.7)	5092 (45.5)	1476 (57.5)
Unknown	12,581	216	143	304	44
Person-years of follow-up					
Mean (SD)	6.8 (4.9)	6.9 (5.1)	8.2 (5.4)	6.6 (4.9)	7.3 (5.2)
Median (IQR)	4 (2–6)	3 (2–6)	4 (2–6)	3 (2–5)	4 (2–6)
Outcome status					
Alive	352,200 (76.2)	7138 (84.8)	6339 (80.7)	9401 (84.0)	1935 (75.3)
Died (all causes)	109,805 (23.8)	1278 (15.2)	1519 (19.3)	1792 (16.0)	634 (24.7)
Breast cancer-specific	46,116 (42.0)	712 (55.7)	554 (36.5)	1061 (59.2)	288 (45.4)
Cardiovascular	25,527 (23.2)	207 (16.2)	381 (25.1)	272 (15.2)	140 (22.1)

	Korean (n = 2755)	Vietnamese (n = 2410)	Asian Indian and Pakistani (n = 2908)	Pacific Islander^a^ (n = 928)	Other Asian^a^ (n = 5494)
Age at index breast cancer					
Mean (SD)	53.3 (11.9)	52.6 (12.2)	53.4 (12.8)	55.0 (12.0)	54.0 (12.5)
Median (IQR)	52 (45–62)	51 (44–60)	53 (44–62)	55 (46–63)	52 (45–62)
20–49 years	1173 (42.6)	1078 (44.7)	1180 (40.6)	318 (34.3)	2220 (40.4)
50–64 years	1060 (38.5)	910 (37.8)	1127 (38.8)	402 (43.3)	2124 (38.7)
65–74 years	372 (13.5)	286 (11.9)	434 (14.9)	150 (16.2)	750 (13.7)
75–85 years	135 (4.9)	115 (4.8)	144 (5.0)	51 (5.5)	345 (6.3)
85+ years	15 (0.5)	21 (0.9)	23 (0.8)	7 (0.8)	55 (1.0)
Birthplace					
U.S.	124 (4.5)	24 (1.0)	135 (4.6)	82 (8.8)	543 (9.9)
Non-U.S.	1792 (65.0)	1637 (67.9)	1525 (52.4)	488 (52.6)	1333 (24.3)
Unknown	839	749	1248	358	3618
Marital status					
Single or never married	275 (10.0)	396 (16.4)	143 (4.9)	110 (11.9)	667 (12.1)
Married or domestic partner	2411 (87.5)	1947 (80.8)	2699 (92.8)	793 (85.5)	4650 (84.6)
Unknown	69	67	66	25	177
AJCC stage					
I	1270 (46.1)	1071 (44.4)	1195 (41.1)	341 (36.7)	2670 (48.6)
II	1085 (39.4)	968 (40.2)	1192 (41.0)	395 (42.6)	2202 (40.1)
III	400 (14.5)	371 (15.4)	521 (17.9)	192 (20.7)	622 (11.3)
Hormone receptor status					
ER(+)/PR(+)	1525 (55.4)	1302 (54.0)	1722 (59.2)	606 (65.3)	3330 (60.6)
ER(+)/PR(−)	263 (9.5)	258 (10.7)	269 (9.3)	80 (8.6)	592 (10.8)
ER(−)/PR(+)	55 (2.0)	53 (2.2)	41 (1.4)	13 (1.4)	76 (1.4)
ER(−)/PR(−)	620 (22.5)	493 (20.5)	614 (21.1)	170 (18.3)	916 (16.7)
Missing	292	304	262	59	580
Tumor size					
<2 cm	2209 (80.2)	1922 (79.8)	2348 (80.7)	709 (76.4)	4671 (85.0)
≥2 cm	537 (19.5)	473 (19.6)	542 (18.6)	209 (22.5)	806 (14.7)
Missing	9	15	18	10	17
Lymph node status					
Negative	1766 (64.1)	1560 (64.7)	1749 (60.1)	538 (58.0)	3696 (67.3)
Positive	989 (35.9)	848 (35.2)	1157 (39.8)	390 (42.0)	1797 (32.7)
Missing		2	2		
Surgery					
Radical or total mastectomy	1376 (49.9)	1391 (57.7)	1425 (49.0)	459 (49.5)	2767 (50.4)
Breast-conserving surgery	1235 (44.8)	940 (39.0)	1386 (47.7)	427 (46.0)	2569 (46.8)
Unspecified surgery	144	79	97	42	158
Radiation					
No	1345 (48.8)	1325 (55.0)	1288 (44.3)	442 (47.6)	2897 (52.7)
Yes	1347 (48.9)	1027 (42.6)	1539 (52.9)	452 (48.7)	2397 (43.6)
Unknown	63	58	81	34	200
Person-years of follow-up					
Mean (SD)	6.2 (4.7)	6.1 (4.6)	5.3 (4.2)	5.2 (4.3)	12 (0.2)
Median (IQR)	3 (2–5)	3 (2–5)	3 (1–4)	2 (1–4)	6 (0.1)
Outcome status					
Alive	2430 (88.2)	2088 (86.6)	2564 (88.2)	751 (80.9)	5012 (91.2)
Died (all causes)	325 (11.8)	322 (13.4)	344 (11.8)	177 (19.1)	482 (8.8)
Breast cancer-specific	219 (67.4)	211 (65.5)	216 (62.8)	101 (57.1)	299 (5.4)
Cardiovascular	33 (10.2)	23 (7.1)	40 (11.6)	20 (11.3)	57 (1.0)

^a^Pacific Islander includes women classified in SEER as Micronesian, Chamorran, Guamanian NOS, Polynesian NOS, Tahitian, Samoan, Tongan, Melanesian NOS, Fiji Islander, New Guinean and Other Pacific Islander; Other Asian women includes women classified in SEER as Thai, Laotian, Hmong, Kampuchean and Other Asian

**Fig. 1:**
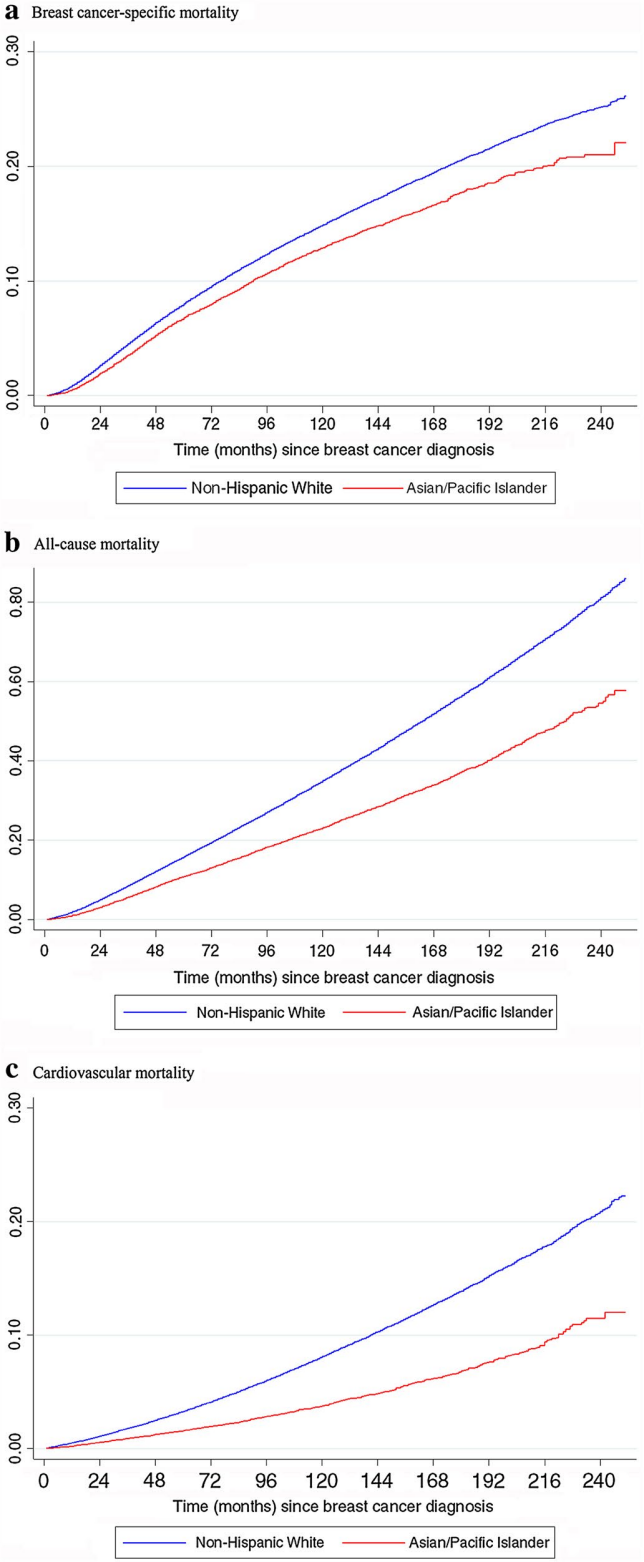
Age-adjusted cumulative hazards of mortality among Non-Hispanic White and Asian/Pacific Islander women. **a** Breast cancer-specific mortality, **b** all-cause mortality, **c** cardiovascular mortality

**Fig. 2:**
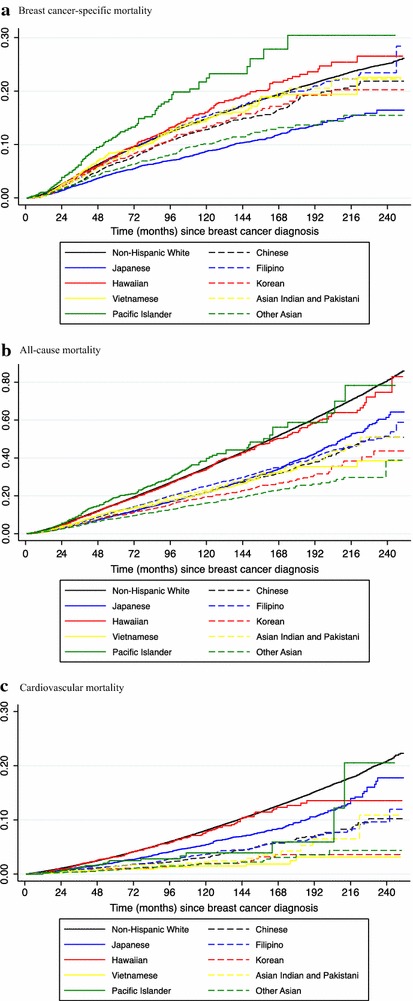
Age-adjusted cumulative hazards of mortality by Asian/Pacific Islander ethnicity. **a** Breast cancer-specific mortality, **b** all-cause mortality, **c** cardiovascular mortality

Age-adjusted cumulative hazards of mortality among NHW and API women with BC are shown in Fig. 1 with the cumulative hazards disaggregated by API ethnicity shown in Fig. 2. Compared to NHW women, API women with BC had significantly lower risks of breast cancer-specific mortality (HR 0.88, 95 % CI 0.84–0.93), cardiovascular mortality (HR 0.77, 95 % CI 0.71–0.83) and all-cause mortality (HR 0.82, 95 % CI 0.80–0.85). These associations differed by API ethnic group with some women having risk of breast cancer mortality similar to NHW women (Filipino, HR 0.93, 95 % CI 0.86–1.00; Hawaiian, HR 1.01, 95 % CI 0.89–1.17) and Pacific Islander women having significantly higher risk of breast cancer mortality (HR 1.44, 95 % CI 1.17–1.78). Hawaiian women had higher risk of cardiovascular mortality (HR 1.43, 95 % 1.17–1.75) and Pacific Islander women had higher risk of all-cause mortality (HR 1.53, 95 % CI 1.30–1.75) compared to NHW women (Table 3).

**Table 3:** Risk of all-cause, breast cancer-specific and cardiovascular mortality among Asian/Pacific Islander women compared to Non-Hispanic White women

	Breast cancer-specific mortality				Cardiovascular mortality				All-cause mortality			
	Age/stage-adjusted^a^		Multivariable-adjusted^b^		Age/stage-adjusted^a^		Multivariable-adjusted^b^		Age/stage-adjusted^a^		Multivariable-adjusted^b^	
	HR	(95 % CI)	HR	(95 % CI)	HR	(95 % CI)	HR	(95 % CI)	HR	(95 % CI)	HR	(95 % CI)
Non-Hispanic White	Ref.		Ref.		Ref.		Ref.		Ref.		Ref.	
All Asian/Pacific Islander women	0.93	(0.89–0.96)	*0.88*	*(0.84*–*0.93)*	*0.76*	*(0.71*–*0.81)*	*0.77*	*(0.71*–*0.83)*	*0.86*	*(0.83*–*0.88)*	*0.82*	*(0.80*–*0.85)*
Chinese	0.92	(0.85–0.99)	0.90	(0.83–0.99)	*0.67*	*(0.58*–*0.77)*	*0.66*	*(0.56*–*0.78)*	*0.80*	*(0.75*–*0.84)*	*0.78*	*(0.75*–*0.84)*
Japanese	*0.72*	*(0.66*–*0.79)*	*0.69*	*(0.63*–*0.77)*	*0.72*	*(0.64*–*0.81)*	*0.71*	*(0.62*–*0.81)*	*0.72*	*(0.68*–*0.76)*	*0.70*	*(0.65*–*0.74)*
Filipino	1.02	(0.96–1.08)	0.93	(0.86–1.00)	0.87	(0.78–0.99)	0.90	(0.78–1.03)	0.94	(0.90–0.99)	*0.86*	*(0.82*–*0.92)*
Hawaiian	1.20	(1.05–1.38)	1.01	(0.89–1.17)	*1.65*	*(1.37*–*2.00)*	*1.43*	*(1.17*–*1.75)*	*1.30*	*(1.19*–*1.43)*	1.09	(0.99–1.20)
Korean	0.97	(0.85–1.10)	0.89	(0.76–1.03)	0.66	(0.47–0.93)	0.68	(0.46–0.99)	0.86	(0.77–0.96)	*0.80*	*(0.71*–*0.91)*
Vietnamese	1.01	(0.88–1.16)	0.84	(0.72–0.98)	*0.52*	*(0.35*–*0.78)*	*0.46*	*(0.28*–*0.76)*	0.95	(0.85–1.06)	*0.81*	*(0.71*–*0.92)*
Asian Indian and Pakistani	0.91	(0.80–1.04)	0.85	(0.73–0.99)	0.84	(0.62–1.15)	0.98	(0.70–1.37)	0.94	(0.84–1.04)	0.91	(0.84–1.04)
Pacific Islander	*1.38*	*(1.13*–*1.68)*	*1.44*	*(1.17*–*1.78)*	1.33	(0.86–2.07)	1.33	(0.83–2.15)	*1.51*	*(1.30*–*1.75)*	*1.53*	*(1.30*–*1.75)*
Other Asian	*0.80*	*(0.72*–*0.90)*	0.95	(0.84–1.08)	*0.56*	*(0.43*–*0.73)*	*0.61*	*(0.45*–*0.83)*	*0.72*	*(0.66*–*0.79)*	*0.83*	*(0.75*–*0.92)*

All estimates are for comparisons of specific Asian/Pacific Islander groups to reference group of Non-Hispanic White women. Italics indicates statistical significance at *P* < 0.05 after Bonferroni correction for multiple comparisons

^a^Model with adjustment for year of diagnosis, SEER registry, age (20–49, 50–64, 65–74, 75–84, 85+ years) and AJCC stage (I, II, III)

^b^Model with adjustment for year of diagnosis, SEER registry, age (20–49, 50–64, 65–74, 75–84, 85+ years), AJCC stage (I, II, III), birthplace (U.S., non-U.S., unknown), marital status (single or never married, married or domestic partner, unknown), hormone receptor status (ER+/PR+, ER+/PR−, ER−/PR+, ER−/PR−, unknown), tumor size (<2, ≥2 cm, unknown), lymph node status (negative, positive, unknown), surgical procedure (radical or total mastectomy, breast-conserving surgery, unspecified surgery), radiation (yes/no)

In analyses comparing U.S. born API women to non-U.S. born API women, no statistically significant differences in risk were observed for breast cancer-specific mortality overall or within any specific API groups. However, U.S. born API breast cancer survivors overall had higher risk of cardiovascular mortality (HR 1.29, 95 % CI 1.08–1.54) and all-cause mortality (HR 1.12, 95 % CI 1.03–1.20) compared to non-U.S. born API breast cancer survivors. While estimates for some specific API groups were suggestive of increased all-cause mortality for U.S. born women (Filipino, HR 1.31, 95 % CI 1.07–1.62; Asian Indian and Pakistani, HR 1.49, 95 % CI 0.79–2.83), these differences in risk of breast cancer-specific, cardiovascular and all-cause mortality by U.S. birthplace were not significant within individual API groups of breast cancer survivors (Table 4).

**Table 4:** Risk of all-cause, breast cancer-specific and cardiovascular mortality among U.S. born Asian/Pacific Islander women compared to non-U.S. born women

	Breast cancer-specific mortality				Cardiovascular mortality				All-cause mortality			
	Age/stage-adjusted^a^		Multivariable-adjusted^b^		Age/stage-adjusted^a^		Multivariable-adjusted^b^		Age/stage-adjusted^a^		Multivariable-adjusted^b^	
	HR	(95 % CI)	HR	(95 % CI)	HR	(95 % CI)	HR	(95 % CI)	HR	(95 % CI)	HR	(95 % CI)
All Asian/Pacific Islander women	1.02	(0.92–1.12)	1.07	(0.96–1.20)	1.23	(1.05–1.44)	*1.29*	*(1.08*–*1.54)*	1.06	(0.99–1.14)	1.12	(1.03–1.20)
Chinese	1.06	(0.83–1.37)	1.01	(0.77–1.33)	1.37	(0.87–2.15)	1.33	(0.81–2.20)	1.03	(0.86–1.25)	1.04	(0.86–1.28)
Japanese	0.97	(0.78–1.22)	1.02	(0.80–1.30)	1.11	(0.81–1.52)	1.04	(0.74–1.48)	0.94	(0.81–1.08)	0.96	(0.82–1.12)
Filipino	1.36	(1.06–1.73)	1.36	(1.04–1.78)	1.00	(0.61–1.66)	0.99	(0.57–1.72)	1.31	(1.08–1.57)	1.31	(1.07–1.62)
Hawaiian	2.41	(0.34–17.4)	2.39	(0.33–17.3)	0.64	(0.15–2.71)	0.97	(0.13–7.38)	1.97	(0.63–6.20)	2.56	(0.63–10.4)
Korean	1.16	(0.62–2.19)	1.14	(0.56–2.32)	0.39	(0.07–2.15)	0.17	(0.02–1.69)	1.16	(0.72–1.89)	1.02	(0.61–1.74)
Vietnamese	0.59	(0.08–4.56)	1.51	(0.20–11.3)	–	–^c^	–	–^c^	2.68	(1.19–6.05)	2.25	(0.78–6.51)
Asian Indian and Pakistani	1.69	(0.97–2.96)	1.49	(0.79–2.83)	0.72	(0.09–5.90)	0.94	(0.11–8.13)	1.52	(0.94–2.46)	1.14	(0.66–1.99)
Pacific Islander	1.99	(0.99–3.97)	1.72	(0.77–3.83)	2.56	(0.54–12.1)	4.27	(0.68–26.7)	1.39	(0.81–2.39)	1.49	(0.82–2.69)
Other Asian	0.44	(0.29–0.66)	0.56	(0.36–0.88)	1.85	(0.85–4.03)	2.06	(0.84–5.10)	*0.43*	*(0.35*–*0.53)*	0.70	(0.50–0.97)

All estimates are among women with documented birthplace in SEER for comparisons within specific Asian/Pacific Islander groups of U.S. born women to reference group of non-U.S. born women. Italics indicates statistical significance at *P* < 0.05 after Bonferroni correction for multiple comparisons

^a^Model with adjustment for year of diagnosis, SEER registry, age (20–49, 50–64, 65–74, 75–84, 85+ years) and AJCC stage (I, II, III)

^b^Model with adjustment for year of diagnosis, SEER registry, age (20–49, 50–64, 65–74, 75–84, 85+ years) and AJCC stage (I, II, III), marital status (single or never married, married or domestic partner, unknown), hormone receptor status (ER+/PR+, ER+/PR−, ER−/PR+, ER−/PR−, unknown), tumor size (<2, ≥2 cm, unknown), lymph node status (negative, positive, unknown), surgical procedure (radical or total mastectomy, breast-conserving surgery, unspecified surgery), radiation (yes/no)

^c^Not enough events to estimate cause-specific mortality

## Discussion

Asian and Pacific Islander women compose a diverse, large and growing population that has traditionally been studied as a single entity. We utilized a large, geographic population-based database to identify differences among this heterogeneous group. Although API women overall have lower breast cancer mortality, differences exist in health outcomes following breast cancer diagnoses depending on national origin (Gomez et al. 2014; Parise and Caggiano 2014; Telli et al. 2011; Yost et al. 2001). Our findings suggest that Pacific Islander women are at increased risk for all-cause and breast-cancer specific mortality relative to NHW women and other API ethnicities. Hawaiian women experienced increased risk for cardiovascular-specific death following breast cancer in comparison to NHW women. We also found that compared to non-U.S. born women, API women born in the U.S. had higher risk of both cardiovascular and all-cause mortality following breast cancer.

The 5-year breast cancer survival rates of API women in the United States and some countries in Asia are over 80 %, with India as an exception (Allemani et al. 2015). The influence of nativity and birthplace on health outcomes were evaluated in other smaller studies (Hedeen et al. 1999; Ziegler et al. 1993; Pineda et al. 2001; Franzini et al. 2001; Gomez et al. 2010). It is hypothesized that acculturation factors related to migration, such as change in diet, could explain differences in breast cancer survival. In a study by Pineda, et al. (Pineda et al. 2001), no differences in survival were found by place of birth among API women with BC in three SEER registries between 1973 and 1994. This study, however, was limited to Chinese, Japanese and Filipino women only. In our study, using more recent years of follow up and information on women from other API ethnicities, we found that API breast cancer survivors born in the U.S. had increased risk for cardiovascular-specific and all-cause mortality. Although not statistically significant, U.S.-born Filipino and Vietnamese women showed a possibly increased risk of all-cause mortality. This suggests that while overall breast cancer survival rates may be comparable, some factors associated with acculturation or U.S. residence may affect other important health outcomes and chronic disease risk such as cardiovascular disease (Ziegler et al. 1993; Lee et al. 2007; Thomas and Karagas 1987).

Our findings are consistent with a growing, yet limited, literature that is available on breast cancer survivors by API ethnicity, including lower risk of cancer-related death and overall mortality in Japanese women in the U.S. (Meng et al. 1997; Hsu et al. 1997). Few studies have examined mortality outcomes among Hawaiian and Pacific Islander women with breast cancer compared to non-Hispanic White women in the U.S. Our study indicates that Pacific Islander and Hawaiian breast cancer survivors had increased mortality risk. Possible factors hypothesized to explain disparities among Pacific Islander and Hawaiian women include differences in dietary fat consumption and physical activity. In a cross-sectional study by Moy, et al. (Moy et al. 2010), native Hawaiian and Pacific Islander women had lower fruit and vegetable consumption, less physical activity and higher prevalence of smoking compared to the general U.S. population. Further, limited access to health care may also be a factor in explaining why the Pacific Islander group may have higher mortality risk. According to the Intercultural Cancer Council in 2003, the only oncology providers for Pacific Islanders were reported to be in Guam (Iammarino and Gribble 2009). Along with other factors, limited access to oncology care could impede appropriate breast cancer treatment and survivorship care.

Differences in health beliefs and practices exist between Non-Hispanic White (NHW) and API women that may influence cancer risk and outcomes. Mammography screening varies considerably by API ethnicity and adherence to screening recommendations in these groups is further impacted by non-U.S. citizen status and having health insurance coverage (Gomez et al. 2007). In a U.S. population-based study (Gomez et al. 2004), API enrollees in a health maintenance organization had lower body mass index (BMI) and were less likely to report alcohol consumption and current or ever smoking. Further, fewer APIs engaged in physical activity compared to their NHW counterparts (Gomez et al. 2004). These characteristics also vary by API ethnicity. Japanese enrollees were more likely to have ever smoked and Filipinos were more likely to have smoked at an earlier age. Chinese and Japanese enrollees had lower BMI, while other API groups had similar or greater BMI compared to non-Hispanic Whites (Gomez et al. 2004). Many of these factors not only affect cancer outcomes but are important to risk of cardiovascular disease and other chronic conditions. With many potential survivorship years post-breast cancer and a likely history of treatment with cardiotoxic chemotherapy and radiation, cardiovascular risk is important to the aging population of API breast cancer survivors (Hooning et al. 2007).

Further research is needed to corroborate these findings and examine cultural factors, biobehavioral mechanisms and health care policies that impact breast cancer survival in different API women. If confirmed, our study has important public health implications. Understanding differences between women of different API ethnicities can help guide tailored interventions to improve outcomes in this heterogeneous group of breast cancer survivors. It is essential that aging women with a history of BC be able to manage the multiple chronic health concerns that follow their last cancer treatment. One way to approach this is by creating more customized survivorship care plans (SCP) for breast cancer survivors (Miller 2008). The purpose of creating breast cancer SCPs is to maximize the health outcomes of these patients during the difficult transition from the end of cancer treatment to care beyond their oncology provider (Hewitt et al. 2006).

According to a review of survivorship care in the cancer literature, less than one half of National Cancer Institute-designated cancer centers provide SCPs to breast cancer survivors. This is partly due to the resources, time and commitment that come with survivorship care planning (Salz et al. 2012). Although these barriers need to be overcome in order for the SCP to be widely adopted, by working towards making these plans more personalized, people may be more likely to utilize them and successfully manage their health post-breast cancer. In a study that explored the experiences of South Asian breast cancer survivors post-treatment (Singh-Carlson et al. 2013), researchers found that this group may have certain spiritual and language-specific support needs that should be taken into consideration when creating a SCP. From our results, we see that certain groups, such as the Hawaiians, may be at greater risk for cardiovascular-specific mortality. This may signal a need to incorporate more regular or targeted surveillance for cardiovascular risk factors in breast cancer survivorship care for some API women.

The distribution of subtypes may differ among ethnic groups, which may be an indicator of breast cancer prognosis and survival (Gomez et al. 2014; Parise and Caggiano 2014; Telli et al. 2011). In addition to tailored interventions and survivorship care plans, these results should spur discussion on ways to improve access to health care and to explore the biology of breast cancer in this heterogeneous group.

Our study has several limitations. We utilized registry-collected data from SEER only, and 36.3 % of the API women did not report their place of birth. Information on other factors that could influence survival after breast cancer including individual-level data measuring dietary intake and socioeconomic status were not available. Other reports have hypothesized that low dietary fat intake among Japanese women partly explains the greater survival observed compared to other racial and ethnic groups in the U.S., although epidemiological studies have not found consistent associations (Holmes et al. 1999; Nomura et al. 1991; Lands et al. 1990).

Despite these limitations, our study has several strengths. We utilized data on a large number of API breast cancer survivors from population-based cancer registries in different regions of the U.S. In evaluating disaggregated ethnic groups of API breast cancer survivors, we report on outcomes among women less represented in the cancer and health disparities literature such as Pacific Islander women.

## Conclusion

In conclusion, aggregation of API women with breast cancer can mask disparities in cancer-specific, cardiovascular- and all-cause mortality. Our findings suggest that specific API populations are at higher risk for mortality after breast cancer diagnosis. With further research and understanding of the API population, these findings should translate to public health practice.

## References

[CR1] Allemani C, Weir HK, Carreira H, Harewood R, Spika D, Wang XS, Bannon F, Ahn JV, Johnson CJ, Bonaventure A, Marcos-Gragera R, Stiller C, Azevedo e Silva G, Chen WQ, Ogunbiyi OJ, Rachet B, Soeberg MJ, You H, Matsuda T, Bielska-Lasota M, Storm H, Tucker TC, Coleman MP, Group CW (2015). Global surveillance of cancer survival 1995–2009: analysis of individual data for 25,676,887 patients from 279 population-based registries in 67 countries (CONCORD-2). Lancet.

[CR2] American Cancer Society (2013). Breast cancer facts & figures 2013–2014.

[CR3] Franzini L, Ribble JC, Keddie AM (2001). Understanding the Hispanic paradox. Ethn Dis.

[CR4] Gomez SL, Kelsey JL, Glaser SL, Lee MM, Sidney S (2004). Immigration and acculturation in relation to health and health-related risk factors among specific Asian subgroups in a health maintenance organization. Am J Public Health.

[CR5] Gomez SL, Tan S, Keegan TH, Clarke CA (2007). Disparities in mammographic screening for Asian women in California: a cross-sectional analysis to identify meaningful groups for targeted intervention. BMC Cancer.

[CR6] Gomez SL, Clarke CA, Shema SJ, Chang ET, Keegan TH, Glaser SL (2010). Disparities in breast cancer survival among Asian women by ethnicity and immigrant status: a population-based study. Am J Public Health.

[CR7] Gomez SL, Glaser SL, Horn-Ross PL, Cheng I, Quach T, Clarke CA, Reynolds P, Shariff-Marco S, Yang J, Lee MM, Satariano WA, Hsing AW (2014). Cancer research in Asian American, Native Hawaiian, and Pacific Islander populations: accelerating cancer knowledge by acknowledging and leveraging heterogeneity. Cancer Epidemiol Biomarkers Prev.

[CR8] Hedeen AN, White E, Taylor V (1999). Ethnicity and birthplace in relation to tumor size and stage in Asian American women with breast cancer. Am J Public Health.

[CR9] Hewitt ME, Ganz PA, Institute of Medicine (U.S.), American Society of Clinical Oncology (U.S.) (2006). From cancer patient to cancer survivor: lost in transition: an American Society of Clinical Oncology and Institute of Medicine Symposium.

[CR10] Holmes MD, Stampfer MJ, Colditz GA, Rosner B, Hunter DJ, Willett WC (1999). Dietary factors and the survival of women with breast carcinoma. Cancer.

[CR11] Hooning MJ, Botma A, Aleman BM, Baaijens MH, Bartelink H, Klijn JG, Taylor CW, van Leeuwen FE (2007). Long-term risk of cardiovascular disease in 10-year survivors of breast cancer. J Natl Cancer Inst.

[CR12] Howlader N, Ries LA, Mariotto AB, Reichman ME, Ruhl J, Cronin KA (2010). Improved estimates of cancer-specific survival rates from population-based data. J Natl Cancer Inst.

[CR13] Hsu JL, Glaser SL, West DW (1997). Racial/ethnic differences in breast cancer survival among San Francisco Bay Area women. J Natl Cancer Inst.

[CR14] Iammarino NK, Gribble G (2009). The Intercultural Cancer Council’s fact sheet series: A tool for public and professional education. J Cancer Educ.

[CR15] Kagawa-Singer M, Pourat N (2000). Asian American and Pacific Islander breast and cervical carcinoma screening rates and healthy people 2000 objectives. Cancer.

[CR16] Lands WE, Hamazaki T, Yamazaki K, Okuyama H, Sakai K, Goto Y, Hubbard VS (1990). Changing dietary patterns. Am J Clin Nutr.

[CR17] Lash TL, Fox MP, Buist DS, Wei F, Field TS, Frost FJ, Geiger AM, Quinn VP, Yood MU, Silliman RA (2007). Mammography surveillance and mortality in older breast cancer survivors. J Clin Oncol.

[CR18] Lee J, Demissie K, Lu SE, Rhoads GG (2007). Cancer incidence among Korean-American immigrants in the United States and native Koreans in South Korea. Cancer Control.

[CR19] Lin SS, Phan JC, Lin AY (2002). Breast cancer characteristics of Vietnamese women in the Greater San Francisco Bay Area. West J Med.

[CR20] Meng L, Maskarinec G, Lee J (1997). Ethnicity and conditional breast cancer survival in Hawaii. J Clin Epidemiol.

[CR21] Miller R (2008). Implementing a survivorship care plan for patients with breast cancer. Clin J Oncol Nurs.

[CR22] Moy KL, Sallis JF, David KJ (2010). Health indicators of Native Hawaiian and Pacific Islanders in the United States. J Community Health.

[CR23] Nomura AM, Marchand LL, Kolonel LN, Hankin JH (1991). The effect of dietary fat on breast cancer survival among Caucasian and japanese women in Hawaii. Breast Cancer Res Treat.

[CR24] Parise C, Caggiano V (2014). Disparities in the risk of the ER/PR/HER2 breast cancer subtypes among Asian Americans in California. Cancer Epidemiol.

[CR25] Pineda MD, White E, Kristal AR, Taylor V (2001). Asian breast cancer survival in the US: a comparison between Asian immigrants, US-born Asian Americans and Caucasians. Int J Epidemiol.

[CR26] Sabatino SA, White MC, Thompson TD, Klabunde CN (2015). Cancer screening test use—United States, 2013. MMWR Morb Mortal Wkly Rep.

[CR27] Salz T, Oeffinger KC, McCabe MS, Layne TM, Bach PB (2012). Survivorship care plans in research and practice. CA Cancer J Clin.

[CR28] Singh-Carlson S, Wong F, Martin L, Nguyen SK (2013). Breast cancer survivorship and South Asian women: understanding about the follow-up care plan and perspectives and preferences for information post treatment. Curr Oncol.

[CR29] Singletary SE, Allred C, Ashley P, Bassett LW, Berry D, Bland KI, Borgen PI, Clark GM, Edge SB, Hayes DF, Hughes LL, Hutter RV, Morrow M, Page DL, Recht A, Theriault RL, Thor A, Weaver DL, Wieand HS, Greene FL (2003). Staging system for breast cancer: revisions for the 6th edition of the AJCC cancer staging manual. Surg Clin North Am.

[CR30] Surveillance Epidemiology and End Results (SEER) Program (2013) Overview of the SEER Program. http://seer.cancer.gov/about/overview.html

[CR31] Telli ML, Chang ET, Kurian AW, Keegan TH, McClure LA, Lichtensztajn D, Ford JM, Gomez SL (2011). Asian ethnicity and breast cancer subtypes: a study from the California Cancer Registry. Breast Cancer Res Treat.

[CR32] Thomas DB, Karagas MR (1987). Cancer in first and second generation Americans. Cancer Res.

[CR33] Thompson CA, Gomez SL, Chan A, Chan JK, McClellan SR, Chung S, Olson C, Nimbal V, Palaniappan LP (2014). Patient and provider characteristics associated with colorectal, breast, and cervical cancer screening among Asian Americans. Cancer Epidemiol Biomarkers Prev.

[CR34] Trinh QD, Nguyen PL, Leow JJ, Dalela D, Chao GF, Mahal BA, Nayak M, Schmid M, Choueiri TK, Aizer AA (2015). Cancer-specific mortality of asian americans diagnosed with cancer: a nationwide population-based assessment. J Natl Cancer Inst.

[CR35] U.S. Census Bureau (2012). United States Bureau of the Census catalog.

[CR36] Varadhan R, Weiss CO, Segal JB, Wu AW, Scharfstein D, Boyd C (2010). Evaluating health outcomes in the presence of competing risks: a review of statistical methods and clinical applications. Med Care.

[CR37] Wu AH, Gomez SL, Vigen C, Kwan ML, Keegan TH, Lu Y, Shariff-Marco S, Monroe KR, Kurian AW, Cheng I, Caan BJ, Lee VS, Roh JM, Sullivan-Halley J, Henderson BE, Bernstein L, John EM, Sposto R (2013). The California Breast Cancer Survivorship Consortium (CBCSC): prognostic factors associated with racial/ethnic differences in breast cancer survival. Cancer Causes Control.

[CR38] Yost K, Perkins C, Cohen R, Morris C, Wright W (2001). Socioeconomic status and breast cancer incidence in California for different race/ethnic groups. Cancer Causes Control.

[CR39] Ziegler RG, Hoover RN, Pike MC, Hildesheim A, Nomura AM, West DW, Wu-Williams AH, Kolonel LN, Horn-Ross PL, Rosenthal JF, Hyer MB (1993). Migration patterns and breast cancer risk in Asian-American women. J Natl Cancer Inst.

